# Sensing Data Concealment in NFTs: A Steganographic Model for Confidential Cross-Border Information Exchange

**DOI:** 10.3390/s24041264

**Published:** 2024-02-16

**Authors:** Ghassan Al-Sumaidaee, Željko Žilić

**Affiliations:** Department of Electrical and Computer Engineering, McGill University, Montréal, QC H3A 0G4, Canada; zeljko.zilic@mcgill.ca

**Keywords:** NFTs, blockchain, steganography

## Abstract

In an era dominated by rapid digitalization of sensed data, the secure exchange of sensitive information poses a critical challenge across various sectors. Established techniques, particularly in emerging technologies like the Internet of Things (IoT), grapple with inherent risks in ensuring data confidentiality, integrity, and vulnerabilities to evolving cyber threats. Blockchain technology, known for its decentralized and tamper-resistant characteristics, stands as a reliable solution for secure data exchange. However, the persistent challenge lies in protecting sensitive information amidst evolving digital landscapes. Among the burgeoning applications of blockchain technology, non-fungible tokens (NFTs) have emerged as digital certificates of ownership, securely recording various types of data on a distributed ledger. Unlike traditional data storage methods, NFTs offer several advantages for secure information exchange. Firstly, their tamperproof nature guarantees the authenticity and integrity of the data. Secondly, NFTs can hold both immutable and mutable data within the same token, simplifying management and access control. Moving beyond their conventional association with art and collectibles, this paper presents a novel approach that utilizes NFTs as dynamic carriers for sensitive information. Our solution leverages the immutable NFT data to serve as a secure data pointer, while the mutable NFT data holds sensitive information protected by steganography. Steganography embeds the data within the NFT, making them invisible to unauthorized eyes, while facilitating portability. This dual approach ensures both data integrity and authorized access, even in the face of evolving digital threats. A performance analysis confirms the approach’s effectiveness, demonstrating its reliability, robustness, and resilience against attacks on hidden data. This paves the way for secure data transmission across diverse industries.

## 1. Introduction

### 1.1. Background

An ever-increasing volume of data is being generated by IoT devices equipped with all kinds of sensors, including those embedded in smart medical devices, monitoring systems for industrial equipment, and environmental sensors for agricultural fields [1,2]. This deluge of data necessitates a streamlined exchange across multiple organizations, as more and more IoT applications become global in scope. Take, for instance, a medical device company manufacturing pacemakers in Montreal that needs to securely share sensor data with a team of doctors in Japan for remote monitoring and analysis. Such cross-border data exchange, while enhancing the ease of collaboration and data utilization, presents formidable challenges in the realms of data security and privacy [3]. These challenges are particularly critical in sectors where sensitive data, in areas such as healthcare, supply chains, and financial services, form the core of their operations and cannot be readily tolerated.

In the context of heightened global interconnectedness and technological progress, the phenomenon of cross-border data sharing has witnessed a rapid surge. The seamless global exchange of data requires trust among entities. However, there is a lack of harmonized frameworks and shared principles between nations [4]. An additional challenge lies in ensuring the secure transmission of shared data from the source to the destination [5].

Blockchain has emerged as a significant enabling technology for trusted and secure data exchange. Derived from cryptographic principles and decentralized architectures, blockchain presents a secure and transparent framework for realizing global applications of IoTs [6]. Its decentralized nature serves to alleviate the risks inherent in centralized control and potential single points of failure, thereby formulating a foundation of trust among entities involved in data exchange [7,8]. Blockchain addresses diverse needs, such as data security, user privacy, and real-time sharing. Its substantial impact on cross-border communication is evident in its ability to overcome challenges associated with traditional systems and methodologies that include time-consuming processes [9].

Since its surge in adoption and popularity around 2016–2017, blockchain technology has demonstrated various applications, encompassing cryptocurrencies, smart contracts, and various emergent uses. One such example among these emerging applications is the rise of non-fungible tokens (NFTs), recognized for their substantial purchase prices, occasionally reaching millions of dollars for ownership [10]. NFTs serve as digital representations of both physical and digital creative works or intellectual property, spanning various domains such as music, digital art, games, gifs, and video clips. The non-fungible nature of NFTs, in contrast to traditional fiat currencies, where each unit is interchangeable, underscores the uniqueness of each token. This distinct quality ensures that each token is a singular entity, representing a specific object. These tokens encapsulate digital information, often in the form of media, with their value calculated in terms of cryptocurrencies [11].

In 2014, the concept of NFTs was first introduced, presenting an attempt to establish a mechanism for artists to assert ownership over digital art [12,13]. However, it was in 2017 that NFTs materialized in the Ethereum blockchain. The Ethereum network, featuring smart contract functionality, facilitated token creation, programming, storage, and trading directly within the blockchain, establishing a more robust and accessible foundation for launching NFT projects. The year 2021 witnessed a significant surge in NFT popularity, as evidenced by a substantial increase in the average daily trading volume of the global NFT market, soaring from USD 180,000 in 2020 to an impressive USD 38 million. Despite this substantial increase, discussions have surfaced regarding a subsequent general decline in 2022 [14,15].

### 1.2. Related Work

The explosive growth of sensor technology and the subsequent decrease in the cost of wearable sensors have paved the way for transformative applications across various industries, with healthcare being one of the most significant beneficiaries. These sensors can be embedded in clothing, watches, and other everyday devices that enable continuous and cost-effective monitoring of the user’s vital signs, such as body temperature, respiratory rate, and heart rate [16]. Within lifestyle and healthcare, sensor-generated data requires privacy and security. Several studies have been proposed to address this need. One such example is the blockchain-based eHealthcare system proposed by [17]. This system leverages wireless body area networks (WBANs) to collect patient data from wearable sensors, which are then securely stored and transmitted using blockchain technology. This approach aims to ensure patient privacy and security through medical data immutability and traceability. Another work, presented by [18], combined IoT, blockchain, and cloud technologies within the medical environment to offer healthcare and tele-medical laboratory services. The platform utilizes sensors to capture vital signs and physiological parameters, transmitting the data through a decentralized platform built upon the Ethereum hybrid network certification system. The system’s efficiency lies in its reduced response time and cost compared to alternative approaches. Focusing on energy and delay-aware healthcare monitoring [19], a blockchain-assisted system for the WBAN-IoT is structured to facilitate three categories of communications: intra-WBAN, inter-WBAN, and beyond-WBAN. It utilizes both body and environment sensors, along with dual sinks for emergency and periodic packet transmission. The system [20] leverages a private Ethereum-based blockchain for communication between wearable sensors and smart devices (smartphones or tablets). IPFS is used to facilitate the distributed storage of health data, while smart contract is used for data management and the association between doctors, patients, and the monitored data.

The scope of NFT applications has transcended their initial association with digital art, now encompassing a variety of sectors including, but not limited to, collectibles, virtual worlds, and supply chain management. For instance, Ref. [21] presented an NFT-based model aimed at enhancing the copyright traceability of off-chain data, contributing to the sustainability of the NFT community. In a different application, Ref. [22] proposed a framework, KD-NFT, that integrates NFT security features with knowledge distillations to address security concerns. This model extends NFT security into machine learning, leveraging blockchain features to recover the training procedure. Another application, presented by [23], introduces an NFT-based framework for managing educational assets in the Metaverse. This framework utilizes blockchain technology to authenticate ownership, safeguard intellectual property, and prevent fraud in educational assets represented as NFTs within the Metaverse. In a distinctive approach, Ref. [24] suggested employing NFTs as an incentive mechanism tied to student assessments. This model reinforces positive behaviour by granting bragging rights and special access based on NFT holdings. Furthermore, Ref. [25] proposed a distributed intelligence networking scheme for autonomous vehicles using NFTs. In this context, NFTs tokenize intelligence, describing it through metadata to enhance understanding and search for intelligence in the complex and trust-lacking Internet of Vehicles. Ref. [26] presented a conceptual model using NFTs to facilitate seamless communication between different blockchain systems. Recognizing the challenges posed by fragmented healthcare blockchain networks in managing health data, the paper proposed a solution that leverages NFT technology to synchronize and secure data exchange across these networks. Ref. [27] proposed an NFT-based solution using blockchain smart contracts, tokenization protocols, and decentralized storage for an efficient medical device traceability and ownership management system. In this model, NFTs serve as digital twins, capturing essential attributes and metadata across the entire life cycle of the medical device, from production to current use.

### 1.3. Contribution

To validate our NFT-based solution for secure data transfer, we leverage real-world sensor data from different participants across diverse age groups and genders performing various physical activities. These real-world data, combined with simulated scenarios, evaluate the effectiveness, security, and privacy of our solution for a range of use cases. While we develop steganography, OTP transfer, and encryption phases, we facilitate the use of standardized tools across ecosystems, which can further streamline collaboration and user data management. This approach enables efficient access and retrieval while ensuring data integrity, security, and potentially even improved ownership and transparency.

This research aims to offer an effective and secure framework for leveraging blockchain technology and NFTs for the secure exchange of sensitive information across diverse sectors. To our knowledge, this work marks the first implementation of NFTs as a mechanism for facilitating cross-border data sharing among diverse businesses. The contributions of this paper include the following:

- We propose using NFTs as carriers for sensitive data in cross-border transfers, ensuring secure and authorized access. Harnessing the unique properties of NFTs, we employ the immutable NFT data as a tamperproof pointer, guaranteeing the authenticity and provenance of the sensitive information. Meanwhile, the mutable NFT data serve as a secure container for the sensitive content itself. This allows for dynamic updates to reflect changes in users’ data, ensuring the information remains current and relevant.
- To address the public accessibility of NFT metadata, we propose leveraging the power of steganography. This enables us to both conceal sensitive information and enhance its protection, offering an effective solution for privacy-conscious users.
- To ensure secure and private user mobility across businesses, we propose an approach that prioritizes user data privacy and integrity during transitions. This objective is achieved through the application of a strong cryptographic technique, specifically OTP, which aligns with our model’s security requirements.
- A structured performance analysis is conducted to assess the practicality and effectiveness of implementing this proposed methodology in real-world scenarios.

### 1.4. Paper Organization

This paper is organized as follows. Section 2 delves into the essential components enabling cross-border data sharing via NFTs. Each component is technically defined and outlined, alongside a detailed scenario illustrating the model’s operational flow. Section 3 showcases the implementation environment and demonstrates the execution of the aforementioned scenario. The feasibility of NFTs as data-sharing mechanisms is evaluated in Section 4, presenting the obtained results and their subsequent discussion. Finally, Section 5 concludes the paper by highlighting key takeaways and outlining promising avenues for future research.

## 2. Materials and Methods

### 2.1. Technical Definitions

This section investigates the elements incorporated in our proposition for operating NFTs as a method for sharing data. The specific functions of each component within the framework will be outlined and explained.

#### 2.1.1. Steganography

**Figure 1:**
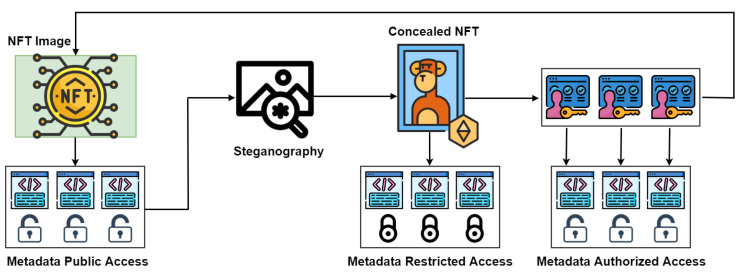
The role of steganography in protecting the NFT public metadata.

Steganography is the art of covertly embedding private information within an ordinary and nonsensitive message, limiting awareness of the concealed content exclusively to the encoder and the intended decoder [28]. Unlike encryption, which focuses on prohibiting data access, steganography is dedicated to masking the existence of the data itself; hence, it is an orthogonal approach. Considering the inherently public accessibility of data within NFTs, our efforts are devoted to securing and safeguarding this information. The employment of steganography techniques serves as a crucial means to achieve this objective. In our specific context, the data involve confidential information linked to various users, necessitating seamless transitions across multiple businesses. The role of steganography in our case is depicted in Figure 1.

The figure illustrates the public nature of NFT metadata. Using steganography on this data can restrict access, granting them only to authorized individuals identified by a specific business.

#### 2.1.2. One-Time Pad (OTP)

**Figure 2:**
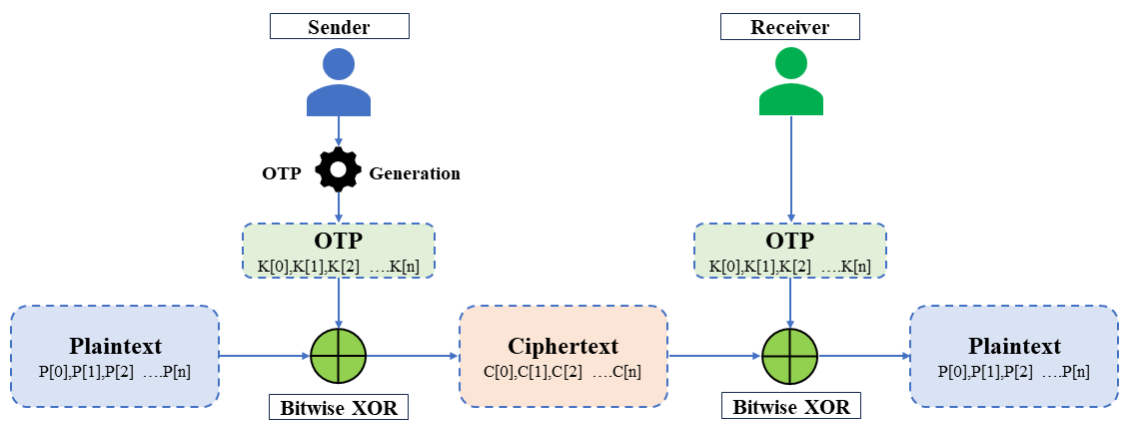
One-time pad encryption and decryption processes.

The one-time pad (OTP) is a cryptographically robust method known for its theoretical invulnerability to ensure confidentiality [29]. It relies on a simple yet highly secure principle using a key matching the message length, characterized by randomness and confidentiality. At its core, OTP involves generating a truly random key, often as a sequence of randomized bits or characters equal to or surpassing the plaintext message length. Illustrated in Figure 2, OTP operations show the encryption process, combining each plaintext element with the corresponding key element through a bitwise XOR operation to produce ciphertext. The key, following the “one-time pad” principle, is for a single use and must be discarded securely afterward. Using this same one-time pad key, the recipient decrypts the message, guaranteeing confidentiality and randomness to reveal the original plaintext. We employed OTP for managing the transfer of steganography passwords between different businesses.

As depicted in the figure, a bitwise XOR operation is performed between the plaintext (representing the password in our case) and a user-specific OTP key. This operation generates ciphertext, which can be securely shared across networks. The receiving network can then use the same OTP key in another XOR operation with the ciphertext to recover the original plaintext (i.e., the password).

#### 2.1.3. NFTs

Cryptocurrencies, mirroring physical currency, demonstrate fungibility, where units within the same currency are interchangeable, such as one Bitcoin for another. In contrast, non-fungible assets, like event tickets, are unique and lack one-to-one exchangeability [30]. NFTs, developed to digitize and trade such assets, represent diverse physical or digital items like art and music. Each NFT possesses a distinct identifier linked to a public blockchain address, ensuring uniqueness among multiple minted NFTs. In our context, we employ NFTs for cross-border data sharing, enabling the secure transfer of sensitive user data across diverse networks. Operating NFTs ensures privacy and security while boosting interoperability among various businesses.

#### 2.1.4. Smart Contract

A smart contract is a specialized program designed to enforce predetermined conditions established by participants within a network. The fundamental role of a smart contract is centred on initiating and overseeing the ledger state within a blockchain framework [31,32]. This responsibility is fulfilled through the processing of transactions submitted by users. Smart contracts operate autonomously, executing predefined actions when certain conditions are met, thereby reducing the need for intermediaries in transactions. These contracts are deployable across diverse blockchain networks, enabling the automated and secure execution of contractual agreements. Additionally, smart contracts ensure transparency and immutability of transactions, as their code is publicly available and stored on the blockchain, providing a verifiable record of all interactions.

### 2.2. Tracing Model Operations

#### 2.2.1. Sensing Data Collection

**Figure 3:**
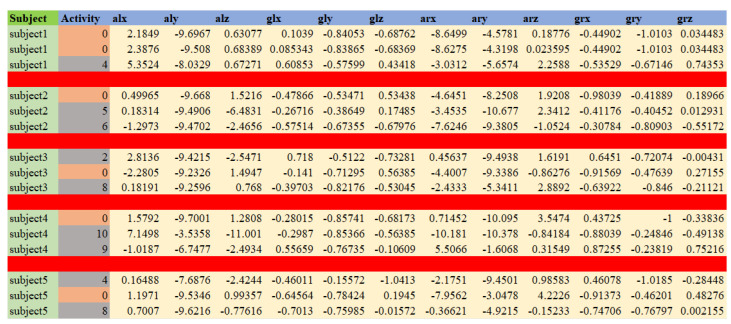
Sample of MHEALTH dataset.

We develop the experiments on the Mobile Health Human Behavior dataset from Kaggle, a prominent online repository for diverse datasets. This dataset comprises body motion and vital signs recordings from ten volunteers of varying demographics performing various physical activities. Sensors positioned on the chest, right wrist, and left ankle captured the movement of different body parts, specifically acceleration, gyroscopic rate of turn, and magnetic field orientation. Data collection employed Shimmer2 wearable sensors. All modalities were recorded at a 50 Hz sampling rate, deemed sufficient for capturing human activity. The activity set encompasses diverse actions, including standing, sitting, relaxing, walking, running, jogging, and more. The MHEALTH dataset, with its diverse sensor data from various individuals performing physical activities, offers a generalizable and privacy-sensitive testing ground for our approach. We consider a subset of these records, generated through sensor data, to be embedded within the NFT image. Figure 3 depicts a sample of these embedded records.

The figure illustrates data pertaining to diverse volunteers engaged in physical activities. Each subject represents a unique volunteer profile. The “activities” field details the 12 distinct physical activities undertaken by these volunteers. Additionally, the remaining fields provide specific values recorded during the conduct of these activities.

#### 2.2.2. Priming NFTs for Use

**Figure 4:**
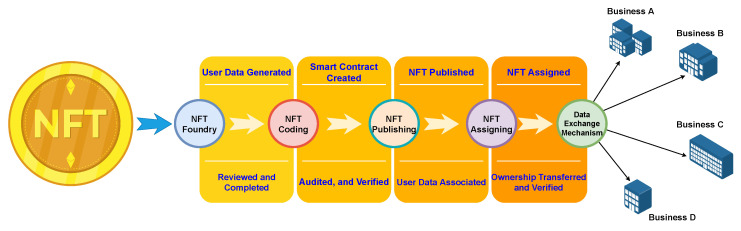
Stages of NFTs evolution towards data exchange accessibility.

Unique NFTs are assigned to users throughout the business ecosystem. Before assignment, multiple steps prepare them for deployment. First, data generation occurs. Each generated NFT holds data for various purposes. In our case, mutable data enables the transfer of sensitive information. These data are carefully reviewed before being generated by each business. Minting NFTs requires a dedicated smart contract. In our scenario, a custom contract is built and deployed on the Ethereum blockchain network. Ethereum, as a pioneer in smart contracts, offers a large developer community, user-friendly language, extensive resources, and streamlined implementation, making it a natural choice despite potential scalability concerns, which we will address through optimization. Typically, the user’s original business’s admin unit is responsible for creating, auditing, and verifying this contract. Upon creation, the contract starts assigning NFTs to users, linking their data to these NFTs. These NFTs with associated data secure users’ sensitive information as they move between businesses. Figure 4 shows the sequential stages through which NFT progresses to achieve the data exchange.

The figure outlines the four stages involved in transforming an NFT into a data exchange mechanism. Initially, the foundry stage focuses on data generation and user verification. Subsequently, the coding stage entails the development and verification of a smart contract. During the publishing stage, the NFT is published, and specific user data is linked to it. Finally, the assignment stage grants the NFT to a user, enabling its function as a data exchange tool.

#### 2.2.3. Scenario

**Figure 5:**
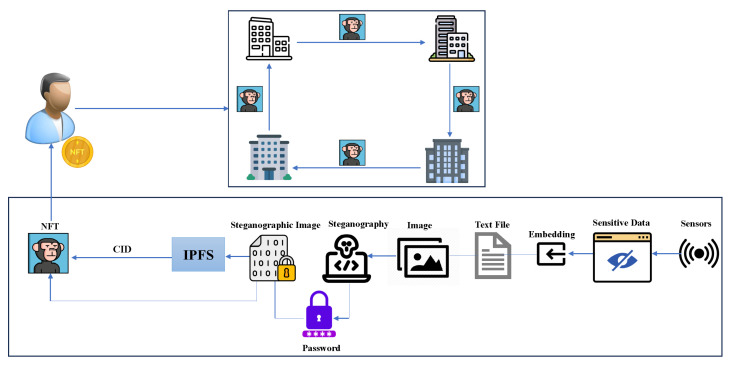
The processes associated with using NFTs for facilitating data exchange.

A consortium of businesses is establishing a secure data exchange system for their users. Sensitive user data samples, collected via sensors (e.g. sourced from the MHEALTH dataset), are stored in a human-readable format, typically a text file. This information is then concealed within an ordinary image $I_{\mathrm{norm}}$ using steganography and creating an encrypted image $I_{\mathrm{enc}}$ with no visible signs of hidden data. Extracting the concealed information requires decrypting $I_{\mathrm{enc}}$ using a password. The NFT creation process enables the incorporation of unique identifiers generated by IPFS, establishing a direct reference to the original data stored within the IPFS system. IPFS (InterPlanetary File System) is a decentralized storage system designed to create a peer-to-peer mechanism for storing and sharing data in a distributed manner. $I_{\mathrm{enc}}$ is then uploaded to IPFS to function as an NFT image. The resulting unique identifier acts as a key within the NFT’s metadata file. This data structure can accommodate a range of user details, such as name, age, and birth date, as determined by the specific business requirements. Additionally, businesses may opt to include other relevant information within the user profile associated with the identifier. The metadata file is also uploaded to IPFS and the obtained unique identifier is used in the NFT smart contract to link $I_{\mathrm{enc}}$ with the metadata. Each user is assigned an NFT, serving as both an ownership mechanism and a holder of sensitive data for cross-border sharing. To transition to a new business, a user’s descriptive data is embedded within the NFT image for transfer. A smart contract on the Ethereum blockchain controls access, ensuring that only authorized administrators can update the NFT’s metadata. The updatable metadata allows changes to the user’s NFT, supporting minting for users to connect with their updated metadata on IPFS using their wallet address. Figure 5 provides the procedures involved in employing NFTs as a means for data exchange.

As shown in the figure, sensitive sensor data is converted into a human-readable format, such as a text file, and prepared for steganographic encoding alongside an image. Both the text file and the image undergo steganographic techniques to embed the data within the image, with added password security. The steganographic image is then uploaded to IPFS, and its unique identifier is embedded within NFT metadata for reference. This results in an NFT image containing sensor data, enabling secure data exchange among businesses through NFT ownership capabilities.

#### 2.2.4. User Mobility

Our model tackles user mobility across diverse businesses, where user data is concealed within steganographic NFT images. As previously mentioned, accessing this data requires a password. Secure and unaltered transmission of this password is crucial, while also actively involving the user. For this, we leverage the power of OTPs.

The first critical step involves the user’s originating network generating an OTP. This OTP acts as a unique binary key, equal or greater in length to the steganography password. It is a random binary sequence of equal length, denoted as (K=K[0],K[1], …, K[L−1]), where (K[i]) represents the (ith) bit. Essentially, the generation process can be summarized as follows:

(1) $$K\left[i\right]\overset{R}{\leftarrow }\left\{0,1\right\}$$

Each bit K[i] is sampled uniformly and independently from 0, 1 using a random process operator $\overset{R}{\leftarrow }$, guaranteeing unbiased selection. The fundamental operation for confidentiality is bitwise XOR(⊕), applied to each corresponding bit pair from *P* and the *K*. The resulting ciphertext (*C*) is obtained by applying (⊕) to each bit, generating a new sequence based on the combined values.

(2) C[i]=P[i]⊕K[i]

where C[i] represents the ith bit of the ciphertext. The OTP, a one-and-done key, is never used twice, ensuring absolute secrecy. The user receives this key to unlock the steganography password in the receiving business or network when the need arises.

With the OTP safely in the user’s hands, the originating network can send the encrypted password *C* through any insecure channel, such as the Internet. Even if compromised, *C* is just random noise without the user’s OTP. The receiving network is helpless without it, as they have the encrypted password, but it means nothing without the user’s decryption code. The user’s exclusive possession of the OTP guarantees password control and security every step of the way, even in the face of potential threats. Algorithm 1 outlines the password transmission process.
**Algorithm 1:** Secure password transmission.**Input**: Steganography password *P*, User’s OTP *K***Output**: Decrypted password *P* for sensitive data access**1** $C_{P}\leftarrow P⊕K$;**2** $C_{P}$→ Transmit (B1 → B2);
**3** B2← Wait;**4** $P\leftarrow C_{P}⊕K$;**5** ⇒ Access sensitive data;

Upon secure reception of the ciphertext *C* by any receiving business, the decryption process requires close collaboration with the user to obtain the steganography password *P*. This decryption process is detailed as follows:

Each bit P[i] of *P* is calculated using a bitwise XOR operation between the corresponding bits of the *C* and the OTP (*K*). This operation can be expressed mathematically as

(3) P[i]=C[i]⊕K[i]fori=0,1,…,L−1

The validation for the decryption process can be represented as follows:

(4) $$F_{\mathrm{Decryption}}\left(C\left[i\right],K\left[i\right]\right)=\left\{\begin{array}{cc} \mathrm{Error} & \mathrm{if}\;C[i]⊕K[i]\neq P[i]\\ P[i] & \mathrm{if}\;C[i]⊕K[i]=P[i] \end{array}\right.$$

The application of the XOR operation serves to reverse the encryption process and retrieve the original password. Algorithm 2 explains the migration scenario between two different businesses.
**Algorithm 2:** User data migration from B_1_ to B_2_.
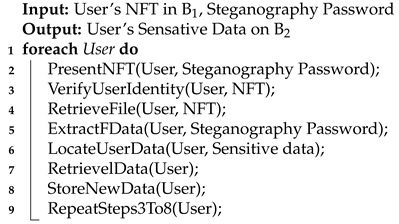


## 3. Setup and Implementation

Within this section, our focus lies on evaluating the performance of the steganography. The objective is to assess the efforts to protect sensitive data associated with individual users. This analysis aids in estimating the reliability and resilience of the technique in concealing confidential information, all while upholding the quality of the cover NFT images. Moreover, it validates the technique’s ability to withstand potential attacks seeking to reveal concealed data. Additionally, it affirms the high similarity between the original and encrypted image, dispelling any uncertainties regarding including sensitive data within the NFT image.

### 3.1. Environment Setup

The evaluation takes place on a local Python 3.8 environment, using specific libraries and tools dedicated to steganography tasks. Development and debugging are carried out within the Pycharm IDE. The analysis is executed on a Windows system operating at 64 bits with 16 GB of RAM and an Intel(R) Core(TM) i7-9750H CPU @ 2.60 GHz. Various parameters and configurations are examined to evaluate steganography performance and identify potential avenues for improvement.

### 3.2. Performance Metrics

In this phase, we assess the performance of steganography, focusing on two key metrics: the structural similarity index (SSIM) and the peak signal-to-noise ratio (PSNR). SSIM provides insights into the quality of hidden information by measuring the similarity between the original and modified images. Higher SSIM values mean seamless embedding of sensitive data, ensuring minimal perceptible alterations to the NFT image’s visual appearance. On the other hand, PSNR quantifies the level of distortion introduced during the steganography process, offering an overall evaluation of the modified image’s fidelity. Preserving the accuracy and reliability of the NFT image is paramount, as any distortion may compromise the privacy and integrity of the embedded data.

SSIM extracts three essential characteristics from an image: luminance, contrast, and structure. The luminance attribute is calculated by averaging all pixel values [33]. In mathematical terms, this computation can be expressed as

(5) $$\bar{\kappa }=\frac{1}{N}\sum_{i=1}^{N}\kappa_{i}\;$$

Here, $\kappa_{i}$ denotes the pixel value of the image κ, and *N* signifies the total count of pixel values within the image. As mentioned earlier, SSIM evaluates similarities between images by considering their luminance, contrast, and structure. Therefore, a comparison function is needed to assess two given images based on their luminance. This luminance comparison function can be mathematically expressed as follows:

(6) $$l\left(\kappa ,\nu \right)=\frac{2\bar{\kappa }\bar{\nu }+\eta_{1}}{{\bar{\kappa }}^{2}+{\bar{\nu }}^{2}+\eta_{1}}$$

Here, $\eta_{1}$ serves as a crucial numerical constant ensuring stability by preventing division by zero. The computation of the contrast feature entails determining the standard deviation of all pixel values. Mathematically, the contrast can be expressed as follows:

(7) $$\sigma ={\left(\frac{1}{N-1}\sum_{i=1}^{N}{\left(\kappa_{i}-\bar{\kappa }\right)}^{2}\right)}^{\frac{1}{2}}$$

Just as with luminance, the contrast comparison function for images κ and ν can be mathematically expressed as follows:

(8) $$c\left(\kappa ,\nu \right)=\frac{2\sigma_{\kappa }\sigma_{\nu }+\eta_{2}}{\sigma_{\kappa }^{2}+\sigma_{\nu }^{2}+\eta_{2}}$$

The third attribute extracted by *SSIM* is known as structure. It entails a process where the input image undergoes division by its standard deviation, leading to the creation of a normalized image with a unit standard deviation. This normalization step is crucial to ensure that the resultant image maintains a unit standard deviation, thereby enhancing the reliability of comparisons. The mathematical representation for the structure of the κ image can be expressed as follows:

(9) $$K=\frac{\kappa -\bar{\kappa }}{\sigma }$$

Just as with both luminance and contrast, the structure comparison function for κ and ν images can be mathematically expressed as follows:

(10) $$S\left(\kappa ,\nu \right)=\frac{\sigma_{\kappa \nu }+\eta_{3}}{\sigma_{\kappa }\sigma_{\nu }+\eta_{3}}$$

$\sigma_{\kappa \nu }$ can be defined as follows:

(11) $$\sigma_{\kappa \nu }=\frac{1}{N-1}\sum_{i=1}^{N}\left(\kappa_{i}-\bar{\kappa }\right)\left(\nu_{i}-\bar{\nu }\right)$$

Then the SSIM score can be expressed as follows:

(12) $$S\left(\kappa ,\nu \right)={\left[l\left(\kappa ,\nu \right)\right]}^{\alpha }{\left[c\left(\kappa ,\nu \right)\right]}^{\beta }{\left[s\left(\kappa ,\nu \right)\right]}^{\gamma }$$

Using parameters α, β, and σ allows for the precise adjustment of the relative importance of the three aforementioned features, each assigned a value >0. To streamline the expression, a simplification can be made by assuming that α=β=γ=1, and $\eta_{3}=\eta_{2}/2$. This simplification results in a specific formulation of the SSIM index:

(13) $$SSIM\left(\kappa ,\nu \right)=\frac{\left(2\bar{\kappa }\bar{\nu }+\eta_{1}\right)\left(2\sigma_{\kappa \nu }+\eta_{2}\right)}{\left({\bar{\kappa }}^{2}+{\bar{\nu }}^{2}+\eta_{1}\right)\left(\sigma_{\kappa }^{2}+\sigma_{\nu }^{2}+\eta_{2}\right)}$$

PSNR relies on the mean squared error (MSE) as a foundational element. Both PSNR and MSE stand out as significant metrics broadly used for evaluating and assessing image quality. The process of determining the PSNR value begins with the measurement of MSE for a specific original and distorted image. Mathematically, the MSE is calculated as follows:

(14) $$MSE=\frac{1}{mn}\sum_{i=0}^{m-1}\sum_{j=0}^{n-1}{\left[\kappa \left(i,j\right)-\nu \left(i,j\right)\right]}^{2}$$

κ(i,j) denotes the original image, ν(i,j) stands for the distorted image, and m,n represent the number of rows and columns in the image. Once the MSE is calculated, determining the PSNR value becomes straightforward through this mathematical equation:

(15) $$PSNR=10\log_{10}\left(\frac{MAX^{2}}{MSE}\right)$$

Here, MAX symbolizes the maximum possible pixel value within the image. When representing pixels with 8 bits per sample, this value is commonly set to 255. A smaller MSE value signifies a reduced level of error, consequently resulting in a higher PSNR value. This higher PSNR value serves as an indicator of enhanced image quality [34].

### 3.3. Parameter Settings and Configurations

In this section, we explore the effects of the key parameters on the efficiency of the steganographic NFT image. Our examination centres on examining how these parameters impact the predefined metrics mentioned previously. The parameters encompass the embedding algorithm, data size, image type and size, password complexity, and encryption mode.

#### 3.3.1. Embedding Algorithm

The choice of the embedding algorithm is a key factor that significantly influences the steganography technique. Although our current configuration uses the least significant bit (LSB) technique, we explore alternative embedding algorithms, including discrete cosine transform (DCT) and discrete wavelet transform (DWT). Our analysis extends to understanding their impact on steganography performance. While LSB conceals data by modifying the least significant bit of pixel values with minimal visual alterations, DWT analyzes image frequency components, enabling data embedding in various frequency bands. DCT, on the other hand, facilitates hiding data in less perceptually significant regions by transforming the image into frequency coefficients. These algorithms present diverse trade-offs in terms of data hiding, resistance to attacks, and image quality.

#### 3.3.2. Data Size

Our objective during this phase is to assess the impact of varying data sizes on the performance of steganographic images used for NFTs. We will specifically embed user-sensitive data provided by the MHEALTH dataset. Since the data are stored in a text file, we will evaluate three distinct file sizes. Initially, we will embed a small piece of sensitive data, resulting in a 1 kB file size. Next, we will increase the file size to 10 kB by incorporating more sensitive data. Finally, we will further expand the volume of descriptive data to investigate the possibility of embedding larger data, resulting in a 20 kB file size. This investigation seeks to establish a clear understanding of the relationship between data size and steganographic image performance.

#### 3.3.3. Image Type and Size

To evaluate the impact of image format on steganographic image quality in NFTs, we conduct an experiment involving two widely used formats: JPEG and PNG. The selection of the image format can impact the visual quality of the steganographic data concealed within the NFT image. Analyzing both formats can assist in providing insights into optimizing steganographic image quality and security within the NFT ecosystem.

#### 3.3.4. Password Complexity

To further enhance the security of sensitive data concealed using steganography, this experimental phase is dedicated to examining the relationship between password strength and its impact on steganographic image integrity. We categorize passwords into three different levels: weak, moderate, and strong (complex). Each password category is subjected to a series of steganography code tests to assess its influence on the steganographic image’s quality and resilience against unauthorized access.

#### 3.3.5. Encryption Mode

To accommodate data larger than its native 128-bit block size, the Advanced Encryption Standard (AES) employs various block cipher modes. These modes define how AES encrypts or decrypts data by treating them as individual blocks of the same size. AES supports multiple block cipher modes, including the Electronic Code Book (ECB), Cipher Block Chaining (CBC), Cipher Feedback (CFB), Output Feedback (OFB), Counter (CTR), and Galois/Counter (GCM) modes. In our experimental setup, we evaluate the impact of each of these modes on steganography performance.

## 4. Results and Discussion

In this section, we present the outcomes derived from the conducted parameters outlined earlier. Additionally, we analyze their influence on the steganographic NFT image and assess their implications for the efficacy of the presented concept in facilitating cross-border data sharing.

### 4.1. Impact of Embedding Algorithm

A comparison of various embedding algorithms for steganography within our model is presented in Table 1. Among the tested algorithms, LSB outperformed DWT and DCT in terms of image quality, as evidenced by its superior SSIM and PSNR values. This indicates that LSB can effectively embed sensitive information into NFT images while minimizing quality degradation. However, LSB exhibited a higher encryption execution time (0.089 s) compared to DCT (0.022 s) and DWT (0.058 s). Despite this, LSB’s decryption execution time (0.025 s) remained reasonable.

Based on the observed performance, we opted for the LSB algorithm for embedding data into NFT images. This decision was driven by LSB’s superior image quality, as measured by SSIM and PSNR, which were critical considerations for us, along with its acceptable execution speed.

### 4.2. Impact of Data Size

Our findings, as illustrated in Table 2, reveal a negative correlation between embedded data size and PSNR value. This inverse relationship stems from the intensifying distortion inflicted upon the cover image as the data payload expands within it. Similarly, the execution time for encryption and decryption exhibits a proportional increase with the burgeoning embedded data size. This escalating demand on computational resources arises from the necessity to process a larger volume of data during both encryption and decryption. In our specific case, where we solely embedded some sensitive information for each user, a data size of 10 kB proved to be the optimal point of balance. This selection strikes a delicate balance between preserving satisfactory SSIM and PSNR values, maintaining feasible execution times for encryption and decryption, and ensuring the NFT image retains high visual fidelity.

### 4.3. Image Type

Table 3 demonstrates that PNG images outperform their JPEG counterparts in terms of PSNR values. This disparity stems from the inherent lossless nature of PNGs, ensuring minimal to no data degradation during the steganographic process. Conversely, JPEGs are inherently lossy, leading to data loss during embedding and compromising PSNR values. Encryption and decryption execution time, however, paints a different picture. JPEG images exhibit significantly shorter processing times compared to PNGs. This can be attributed to the inherent simplicity of JPEGs, which translates to reduced computational demands during encryption and decryption operations.

Considering these trade-offs, our decision leaned towards adopting PNG images for embedding sensitive data into NFT images. This choice was driven by the superior PSNR performance of PNGs and their ability to maintain acceptable encryption and decryption execution times despite their higher computational requirements.

### 4.4. Impact of Password Complexity

Table 4 reveals the minimal influence of password complexity on both NFT image quality and encryption/decryption times. Regardless of the chosen mode (weak, moderate, or strong), image quality remains consistently high (SSIM of 0.999) with only negligible variations in PSNR. Similarly, encryption and decryption execution times exhibit remarkable uniformity across all password complexities, differing only by milliseconds. This suggests that, within the confines of this steganographic setup, prioritizing robust security measures should take precedence over password complexity. Image fidelity and computational efficiency remain unperturbed by password strength, rendering complex passwords unnecessary for maintaining acceptable image quality. Therefore, we opted for a strong password, acknowledging its alignment with best security practices while still achieving an acceptable PSNR value.

### 4.5. Impact of Encryption Mode

The results presented in Table 5 reveal minimal influence of encryption mode on NFT image quality. All tested modes show remarkably high SSIM values of 0.999, and PSNR values hover around a consistent 82 dB, demonstrating negligible quality degradation across the board. Furthermore, encryption and decryption execution times show comparable performance with slight, millisecond-range variations across all modes. This consistency suggests that image quality remains virtually unaltered regardless of the chosen encryption mode.

Based on these findings, we opted for CFB mode in our implementation due to its superior PSNR performance. While other modes achieved statistically comparable results, CFB’s slight edge in preserving image fidelity ultimately informed our decision.

### 4.6. Discussion

Our research explored hiding users’ sensitive information in NFT images using steganography, a technique for concealing data without raising suspicion. We found that it is possible to embed critical data with a surprisingly minuscule impact on image quality, hence offering a solution for secure cross-border data sharing. We found the best hiding methods, balancing how much information to hide while keeping the picture quality high. We also considered different image formats and passwords, finding that such parameters are the key to maintaining security. While different encryption methods worked similarly, we selected the one that performed slightly better. This opens doors for exciting possibilities for securely sharing sensitive information, adding hidden features to NFTs, and even embedding secret messages in artwork. However, potential security risks require ongoing awareness and responsible use. Further research is needed to explore wider applications while considering legal and ethical implications.

### 4.7. Comparison with Related Works

This section presents a comparative assessment of our model against existing cross-border solutions. These solutions employ novel technologies to address diverse challenges, including optimizing international business strategies, ensuring secure product tracking, and facilitating collaborative logistics management. Furthermore, secure data sharing platforms with built-in accountability are also proposed, highlighting the potential of technology to create cross-border operations. We evaluate each solution based on a set of predefined metrics, indicated with “Yes” if the solution fulfills the metric, “Yes/No” if partially fulfilled, and “No” if not. Table 6 summarizes the comparison, highlighting the strengths and limitations of our approach relative to other published work and established systems.

## 5. Conclusions

The challenge of secure and confidential data exchange has not received the level of attention it deserves in existing research, with proposed solutions often plagued by practical complexities or insecure mechanisms. Non-fungible tokens (NFTs), a burgeoning blockchain application, have recently gained widespread interest for their potential beyond their traditional association with digital art and collectibles. This paper contributes to ongoing NFT exploration by investigating their potential for embedding sensing data within NFTs, enabling confidential and cross-border data exchange.

To achieve this, the proposed solution leverages both the immutable and mutable attributes of NFTs. Immutable data serve as a secure pointer and establish data ownership, while mutable data act as a secure container for sensitive information. Steganography safeguards the confidentiality of data embedded within the NFT, addressing the inherently public nature of NFT metadata. User mobility across multiple businesses is maintained through the employment of a robust cryptographic technique, in our case, a one-time password (OTP) that prioritizes user data privacy and integrity during transitions.

Our experiments demonstrated several insights into embedding sensor data within NFTs using steganography. The type of embedding algorithm was the most crucial factor impacting imperceptibility, with LSB offering near-flawless visual quality (SSIM = 0.99, PSNR > 85) but slower execution times, while DCT and DWT sacrificed some imperceptibility with lower SSIM (<0.99) and lower PSNR (<33 dB). Data size also mattered, as larger data loads decreased imperceptibility and significantly increased execution times. Password complexity and encryption mode had negligible effects on both visual quality and performance. Image type played a minor role, with JPEGs impacting imperceptibility compared to PNGs. To expand the applicability of our findings, future work could consider analyzing additional formats like BMP or TIFF for a more comprehensive understanding.

Our research paves the way for secure data exchange using NFTs, but practical hurdles for wider adoption remain. Standardized tools are crucial for enhancing the adaptability of the proposed solution and facilitating user-friendly and effortless adoption. Future work could aim for interoperable SDKs for user-friendly OTP key generation, encryption/decryption, and steganographic operations, integrated within existing NFT platforms. Open-source collaboration can accelerate development and adoption. Furthermore, standardized data formats, integration with decentralized identity solutions, and advanced steganography techniques hold promise for further enhancing security and the user experience.

## Figures and Tables

**Table 1:** Results of embedding algorithms and their corresponding parameters.

	PSNR (dB)	SSIM	Encryption Execution Time (s)	Decryption Execution Time (s)
**LSB**	85.36	0.99	0.089	0.025
**DCT**	32.92	0.97	0.022	0.001
**DWT**	30.72	0.94	0.058	0.007

**Table 2:** Results of data sizes and their corresponding parameters.

	PSNR (dB)	SSIM	Encryption Execution Time (s)	Decryption Execution Time (s)
**1 KB**	82.009	0.999	0.053	0.021
**10 KB**	62.872	0.999	0.163	0.119
**20 KB**	59.747	0.999	0.273	0.209

**Table 3:** Results of image types and their corresponding parameters.

	PSNR (dB)	SSIM	Encryption Execution Time (s)	Decryption Execution Time (s)
**JPEG**	79.669	0.999	0.059	0.021
**PNG**	93.845	0.999	0.262	0.054

**Table 4:** Results of password modes and their corresponding parameters.

	PSNR (dB)	SSIM	Encryption Execution Time (s)	Decryption Execution Time (s)
**Weak**	81.941	0.999	0.052	0.029
**Moderate**	82.483	0.999	0.053	0.020
**Strong**	81.836	0.999	0.054	0.020

**Table 5:** Results of encryption mode and their corresponding parameters.

	PSNR (dB)	SSIM	Encryption Execution Time (s)	Decryption Execution Time (s)
**CBC**	82.027	0.999	0.053	0.019
**CFB**	82.379	0.999	0.051	0.018
**CTR**	82.070	0.999	0.052	0.019
**OFB**	82.050	0.999	0.049	0.017
**GCM**	81.949	0.999	0.051	0.020
**ECB**	82.035	0.999	0.050	0.018

**Table 6:** Comparison between our model and other solutions.

Metric	[35]	[36]	[4]	[37]	Our Model
**Ownership**	No	No	Yes	No	Yes
**Privacy**	Yes	No	Yes	No	Yes
**Decentralization**	Yes/No	Yes/No	Yes/No	No	Yes
**Immutability**	Yes	Yes	Yes	No	Yes
**Interoperability**	No	No	No	No	Yes
**Robustness Against Attacks**	No	Yes	Yes	No	Yes
**User-Friendly Deployment**	No	No	No	No	No
**Analysis**	Yes	Yes	Yes	Yes	Yes

## Data Availability

The data presented in this study are available upon request from the corresponding author.
